# Ischemia-Induced Neurodegeneration in Glaucoma: Mechanistic Insights and Translational Opportunities for Psychoplastogen-Based Therapies

**DOI:** 10.3390/ph19020316

**Published:** 2026-02-14

**Authors:** Petra Dolenec, Goran Pelčić, Kristina Pilipović, Jasenka Mršić-Pelčić, Anja Harej Hrkać

**Affiliations:** 1 Department of Basic and Clinical Pharmacology and Toxicology, Faculty of Medicine, University of Rijeka, 51000 Rijeka, Croatia; petra.dolenec@uniri.hr (P.D.); kristina.pilipovic@uniri.hr (K.P.); jasenka.mrsic.pelcic@uniri.hr (J.M.-P.); 2 Clinics for Ophthalmology, Clinical Hospital Centre Rijeka, 51000 Rijeka, Croatia; goran.pelcic@medri.uniri.hr

**Keywords:** glaucoma, ischemia, neurodegeneration, psychoplastogens, psychedelics

## Abstract

Glaucoma is increasingly recognized as an ischemic neurodegenerative disorder that extends beyond elevated intraocular pressure (IOP) to involve complex vascular, metabolic, and inflammatory mechanisms. Retinal ganglion cells are particularly vulnerable to ischemia–reperfusion injury, oxidative stress, and chronic neuroinflammation, leading to progressive disconnection from central visual pathways. Current therapies primarily target IOP reduction but fail to address ischemia-driven neurodegeneration or to restore lost neuronal connectivity. Ischemia triggers excitotoxicity, oxidative stress, and a maladaptive inflammatory response involving activated microglia and astrocytes, perpetuating neuronal injury and suppressing intrinsic regenerative capacity. Thus, restoring neural plasticity and mitigating neuroinflammation represent key unmet therapeutic needs. Psychoplastogens are a class of compounds capable of rapidly enhancing structural and functional neuroplasticity and have recently emerged as promising multitarget agents. Compounds such as ketamine, psilocybin, N,N-dimethyltryptamine (DMT), and some newly synthesized non-hallucinogenic analogs act through convergent signaling pathways involving BDNF–TrkB–mTOR, promoting dendritic growth, synaptogenesis, and glial modulation. Beyond their neurotrophic effects, psychoplastogens seem to exert potent immunomodulatory actions. In this review we will explore the interplay between ischemia, neurodegeneration, neuroinflammation, and impaired plasticity in glaucoma, integrating mechanistic insights from cerebral ischemia. We discuss emerging preclinical evidence supporting psychoplastogens as neurorestorative and anti-inflammatory agents, propose their potential application in ocular ischemic neurodegeneration, and outline translational challenges for future studies.

## 1. Introduction

Glaucoma is currently defined by the European Glaucoma Society Guidelines (2026) as a chronic, progressive optic neuropathy characterised by structural damage to the optic nerve head and corresponding retinal nerve fiber layer loss, associated with functional visual field impairment, with intraocular pressure (IOP) as the most important, but not exclusive, modifiable risk factor [1]. This definition emphasises that glaucoma can develop and progress with or without elevated IOP, reflecting its multifactorial and neurodegenerative nature rather than being caused solely by elevated eye pressure. Epidemiologically, glaucoma affects more than 76 million people worldwide and is widely acknowledged as one of the leading causes of irreversible blindness. This number is expected to exceed 110 million by 2040 due to ageing populations and rising life expectancy [2]. While elevated IOP remains a major risk factor and the primary target of current therapies, it is increasingly evident that pressure-lowering interventions alone are insufficient to halt disease progression or prevent irreversible vision loss in many patients. A growing body of evidence implicates vascular dysregulation, excitotoxicity, mitochondrial dysfunction, neuroinflammation, and impaired axonal transport as central contributors to glaucomatous damage. These processes initiate a cascade of deleterious events that extend beyond the anterior segment of the eye, affecting the entire visual pathway from the retina to the brain and positioning glaucoma within the broader spectrum of ischemia-driven neurodegenerative disorders, sharing pathogenetic parallels with cerebral ischemia and Alzheimer’s or Parkinson’s disease [3,4].

Clinically, glaucoma progresses through a series of distinct stages that cumulatively result in irreversible visual impairment [5]. The disease typically originates in a preperimetric phase, marked by subtle retinal ganglion cell (RGC) loss and optic nerve head remodeling occurring in the absence of detectable visual field abnormalities [6,7,8]. Early astrocytic and glial changes within the optic nerve head, detectable with high-resolution imaging and histopathology, have recently been shown to precede RGC loss and functional deficit, suggesting that structural and cellular vulnerability occurs before visual field changes [9,10,11]. During this early stage, vascular dysregulation, mitochondrial dysfunction, impaired axonal transport, and responses of resident glial cells to mechanical and metabolic stress are already evident, silently predisposing the visual system to subsequent neurodegeneration [12,13,14,15,16]. As glaucoma advances to the moderate stages, ongoing RGC apoptosis leads to the development of localized visual field defects, accompanied by escalating ischemia, oxidative stress, mitochondrial dysfunction, and activation of neuroinflammatory pathways [17,18,19]. During this phase, synaptic disconnection and dendritic simplification increasingly limit endogenous repair mechanisms, yet the visual system retains a degree of experience-dependent and injury-induced plasticity that may be therapeutically exploitable. In advanced and end-stage disease, extensive axonal degeneration, widespread visual field constriction, and profound disruption of both retinal and central visual pathways are observed, reflecting a self-reinforcing cycle of chronic hypoperfusion, excitotoxicity, and sustained inflammation [5,20]. Notably, the later stages of glaucoma are largely refractory to therapeutic intervention, highlighting the critical importance of early disease modification and the development of effective neuroprotective and neurorestorative strategies [21,22].

Current clinical management of glaucoma remains focused on reducing IOP through pharmacological, laser, or surgical approaches. Although pressure-lowering therapies can slow disease progression, many patients continue to experience neurodegeneration and vision loss despite well-controlled IOP [23]. This “IOP-independent” progression highlights the limitations of current treatments and underscores the urgent need for novel neuroprotective and neurorestorative strategies that address the underlying ischemic and inflammatory components of the disease [2].

Vascular dysregulation and chronic hypoperfusion are central contributors to glaucomatous neurodegeneration. Reduced ocular blood flow (OBF) and impaired autoregulation lead to intermittent episodes of ischemia–reperfusion, generating bursts of reactive oxygen species (ROS) and initiating oxidative stress and mitochondrial dysfunction [24,25]. These metabolic disturbances trigger excitotoxicity mediated by excessive glutamate release and calcium overload, resulting in RGC apoptosis and axonal degeneration [26]. Furthermore, ischemic injury disrupts the integrity of the blood–retinal barrier, promoting infiltration of inflammatory mediators and activation of glial cells, which further exacerbate neuronal damage [18,27].

Neuroinflammation is a critical, self-perpetuating component of glaucomatous pathology. Activated microglia and astrocytes, although initially protective, often develop a chronic pro-inflammatory phenotype that sustains the release of cytokines, chemokines, and complement components [17,28]. This maladaptive glial activation amplifies ischemic injury and disrupts neuronal survival signaling and synaptic maintenance [15]. Persistent inflammation creates a degenerative microenvironment marked by synaptic pruning, dendritic retraction, and reduced cellular resilience [29]. Thus, ischemia-induced neuroinflammation forms a mechanistic link between vascular dysfunction and progressive neuronal loss in glaucoma [30].

An emerging concept in glaucomatous neurodegeneration is the loss of neuroplasticity. In the healthy visual system, neural circuits retain a degree of adaptability that enables functional recovery after transient insults. However, ischemia and chronic inflammation suppress intrinsic regenerative mechanisms by downregulating neurotrophic factors such as brain-derived neurotrophic factor (BDNF) and impairing signaling pathways crucial for axonal growth and synaptogenesis [31]. This deficit in neuroplasticity limits the capacity for neuronal repair and reconnection of damaged visual pathways. Restoring this plastic potential has therefore become an appealing therapeutic goal in both ocular and central nervous system (CNS) neurodegeneration [32,33].

In this context, psychoplastogens, a recently defined class of compounds capable of rapidly enhancing structural and functional neuroplasticity, have attracted considerable attention as potential neurorestorative agents [34]. Compounds such as ketamine, psilocybin, N, N-dimethyltryptamine (DMT), and newly developed non-hallucinogenic analogues exert their effects through convergent molecular cascades involving BDNF–tyrosine receptor kinase B (TrkB)–mammalian target of rapamycin (mTOR) signaling [35,36]. Activation of these pathways promotes dendritic growth, synaptic formation, and enhanced neuronal connectivity, processes that are markedly compromised in ischemic and inflammatory conditions [37]. Beyond their neurotrophic actions, psychoplastogens demonstrate significant immunomodulatory and anti-inflammatory properties, including suppression of microglial activation and normalization of glial phenotypes [38,39]. These dual actions, enhancement of plasticity and attenuation of neuroinflammation, suggest that psychoplastogens could address both the degenerative and regenerative aspects of glaucomatous pathology.

Translating psychoplastogen-based therapies from neuropsychiatric and cortical models to ocular ischemic neurodegeneration presents both challenges and opportunities. The retina, as an accessible extension of the CNS, offers a unique platform to study therapeutic options for neurorestoration and to evaluate functional outcomes in vivo [40]. By integrating mechanistic insights from cerebral ischemia, neuroinflammation, and psychoplastogen pharmacology, a new therapeutic framework may emerge, one that not only prevents further RGC loss but also promotes structural and functional recovery within the visual system.

This review will therefore explore the interplay between ischemia, neurodegeneration, neuroinflammation, and impaired plasticity in glaucoma, emphasising the translational potential of psychoplastogen-based interventions to modulate these interconnected processes.

## 2. Mechanisms of Ischemia-Induced Pathological Changes in Glaucoma

### 2.1. Ischemia-Induced Neurodegeneration in Glaucoma

Ischemia-induced neurodegeneration in glaucoma involves multiple interconnected mechanisms. It involves OBF impairment with dysregulated autoregulation, mitochondrial energy failure in RGCs, oxidative stress and glutamate excitotoxicity, as well as ischemia–reperfusion-driven inflammation [30]. These overlapping pathways lead to progressive RGCs loss and optic nerve damage characteristic of glaucoma.

RGCs are known to have high metabolic nature and require continual supply of metabolites and removal of oxidative waste [41]. In all stages of glaucoma and independently of IOP, functional and morphological changes appear in the microvasculature of both the retina and optic nerve head [42]. In glaucoma patients OBF is reduced compared with healthy controls and it is a recognized progressive visual field loss contributing factor [43,44]. In normotensive glaucoma (NTG), OBF is more reduced compared to patients with ocular hypertension due to a combination of factors. Namely, NTG is driven largely by vascular dysregulation as NTG patients often have impaired autoregulation of OBF, vasospasms, systemic hypotension (especially nocturnal dips), and endothelial dysfunction [44]. This predilects the optic nerve to ischemic episodes, leading to lower ocular perfusion pressure and lower OBF. Conversely, in high-IOP glaucoma, blood flow is also reduced, but through a primarily mechanical mechanism as elevated IOP compresses the lamina cribrosa and ocular vessels, resulting in decreased perfusion [45]. Unlike in NTG, this reduction is proportional and predictable, without evidence of vascular dysregulation.

Vascular dysfunction in the retina causes recurrent mild ischemia leading to hypoxia, nutrient deficiency, oxidative stress, and ultimately apoptosis of RGCs [46]. Impaired endothelial function and disrupted nitric oxide signaling destabilize autoregulation and ocular perfusion [47]. Mitochondrial dysfunction further compromises RGCs energy supply, increasing vulnerability to oxidative damage [48]. Ischemia elevates glutamate release, triggering excitotoxicity through N-methyl-D-aspartate (NMDA) receptors-mediated calcium influx and pro-death signaling [49]. Local inflammation, astrocyte activation, and extracellular matrix changes create a neurotoxic environment that amplifies oxidative stress and reperfusion episodes release bursts of reactive oxygen species and inflammatory mediators, sustaining the cycle of RGCs degeneration in glaucoma [48].

### 2.2. Molecular and Cellular Constraints on Retinal Ganglion Cell Survival and Regeneration in Glaucoma

As the RGCs are the part of the CNS, they lack the innate ability to regrow once damaged. There are some key signaling mechanisms underlying RGC death, as well as intrinsic and extrinsic factors that constrain regeneration.

The death of RGCs in glaucoma is rarely caused by a single event. Instead, several interconnected pathways, triggered by mechanical stress, vascular dysregulation, and neurotoxic signaling, converge to trigger programmed cell death. Elevated IOP or ischemia leads to an excessive release and accumulation of glutamate, overstimulation of NMDA and AMPA (α-amino-3-hydroxy-5-methyl-4-isoxazolepropionic acid) receptors, causing a massive influx of calcium, which activates proteases and damages the mitochondria in a process known as excitotoxicity [50,51,52,53]. Because of the RGCs high metabolic demand, they are particularly sensitive to mitochondrial dysfunction, producing high levels of ROS, leading to mitochondrial damage, release of cytochrome c, and activation of caspase-dependent apoptosis [54]. ROS also cause mitochondrial DNA damage and activation of the major pro-apoptotic signaling cascades [55,56]. Most RGC death signals converge on the Bcl-2 family of proteins [57]. Specifically, the activation and translocation of Bax to the mitochondrial membrane is considered the irreversible step that triggers the caspase cascade (caspase-3, -8, -9), leading to cell dismantling. Further on, elevated IOP impairs retrograde transport of essential neurotrophic factors (e.g., BDNF), causing neurotrophin deprivation thus starving the RGCs of these essential pro-survival signals [58].

RGCs in the adult mammalian retina have a poor inherent capacity for axon regrowth, due to several internal constraints. Anti-regenerative transcriptional programs in mature RGCs strongly limit their regenerative capacity. Developmental growth-associated genes such as GAP-43 are downregulated in adulthood, while growth suppressors including KLF-4 and SOCS3 are upregulated, actively repressing axon growth and constraining regenerative transcriptional responses following glaucomatous injury [59,60,61]. In adult RGCs, the chromatin structure around regeneration-associated genes becomes tightly packed (heterochromatin), making it physically impossible for the cell to activate the machinery needed for regrowth [62]. Limited progenitor availability further restricts RGC regeneration in mammals [63]. Unlike non-mammalian vertebrates such as zebrafish, the adult mammalian retina lacks a robust endogenous stem cell pool. Müller glia remain largely quiescent and fail to function as effective neural progenitors, rendering cell replacement an unviable endogenous regenerative mechanism [64].

Beyond internal, there are also extrinsic barriers in the injured optic pathway. They create a strongly inhibitory environment for axon regeneration. Myelin-associated inhibitors, including Nogo-A, MAG, and OMgp, released after myelin breakdown bind receptors such as NgR on RGC growth cones and activate RhoA/ROCK signaling, causing growth cone collapse [65,66]. Concurrently, reactive astrocytes and microglia form a glial scar rich in chondroitin sulfate proteoglycans, creating a physical and chemical barrier [67]. Chronic neuroinflammation, characterized by cytokines such as tumor necrosis factor-α (TNF-α), further reinforces a non-permissive regenerative milieu.

### 2.3. Neuroinflammation in Ischemic Glaucoma

Neuroinflammation is considered to have a significant role in the pathogenesis of glaucoma [48,68]. This process is largely mediated by resident glial cells and is often referred to as retinal glial cell activation [69]. Microglial activation is one of the first events in glaucomatous neurodegeneration following elevated IOP or ischemia [19]. In response to glaucoma-related stress, astrocytes undergo a significant transformation known as reactive astrogliosis, where they become activated and release inflammatory mediators [70]. Müller cells, the principal radial astroglia of the retina, also prominently respond in glaucoma [71]. Neuroinflammatory cascade involves several specific molecular pathways and mediators: astrocytes and microglia recognize and respond to disturbances using pattern recognition receptors which are activated by damage-associated molecular patterns released from damaged cells (including RGCs and glia) [72]. Activated glial cells release a plethora of these mediators, which amplify the inflammatory response and can be neurotoxic, including TNF- α, interleukin(IL)-1β, and IL-6 [73]. In addition, neuroinflammation in glaucoma includes activation of several signaling pathways such as NF-κB (leading to increased production of the aforementioned pro-inflammatory cytokines) [74] and SARM1 (linked to axon degeneration, induced by TNF-α, and related to mitochondrial dysfunction) pathways [75].

While acute injury (like severe ischemia–reperfusion injury or a massive increase in IOP) causes a rapid, protective inflammatory response, chronic glaucoma is characterized by a prolonged, low-level state of inflammation that eventually shifts from being protective to being neurotoxic [76]. While early glial responses have adaptive and reparative features, under the continued stress of glaucoma this adaptive state cannot be maintained and the glial cells (most prominently microglia) become “primed” and shift to a chronic pro-inflammatory and neurodegenerative phenotype [17]. Further on, reactive astrocytes interact with the chronically activated microglia and the microglial-derived signals can lead to the differentiation of astrocytes into the neurotoxic phenotype, which is then capable of facilitating RGCs injury and further amplifying the inflammatory environment [70]. The chronic, low-grade inflammation is not just a side effect: it is rather an interdependent pathogenic process that drives the progressive nature of the disease. This means that even if the primary trigger (e.g., IOP) is lowered, the chronic inflammatory and degenerative machinery may already be established and continue to drive RGC loss [77].

### 2.4. Neural Plasticity and Regenerative Capacity in the Adult Visual System

Functional recovery in glaucoma and other optic neuropathies greatly depends on the balance between neural plasticity and regenerative capacity [78]. Namely, in the adult visual system, high plasticity in the brain’s visual cortex is often counterbalanced by extremely limited regenerative capacity in the retinal and optic nerve tissues. As the retina and optic nerve are part of the CNS, they adhere to the CNS’s fundamental limitations regarding regeneration in adulthood, i.e., glaucoma-caused blindness is irreversible because RGCs do not possess self-renewal or self-repair capacity in the adult state [79,80]. This is caused by intrinsic inhibitory factors (such as anti-regenerative transcriptional factors and the limited progenitor cells) as well as extrinsic environmental barriers (including post-injury myelin and glial scar). In contrast, the visual cortex (V1) of the adult brain retains a significant capacity for neural plasticity in response to retinal damage or sensory deprivation due to processes that include retinotopic map reorganization, adaptation to sensory loss, and homeostatic plasticity [33,81,82].

Ischemia and inflammation, core components of glaucomatous injury, fundamentally impair regenerative potential by creating a toxic, non-permissive environment: ischemia/hypoxia causes direct, rapid death of RGCs and mitochondrial failure, eliminating the cells that need to be regenerated, neuroinflammation creates the glial scar which forms a physical barrier and releases inhibitory molecules that actively repel RGC axons, and the pro-inflammatory cytokines overwhelm pro-survival and pro-regenerative signals thus tipping the balance toward cell death [79].

**Figure 1:**
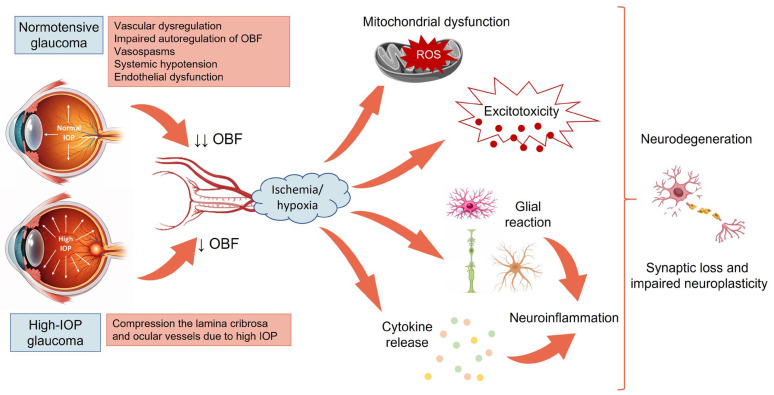
Pathogenic mechanisms leading to neurodegeneration and synaptic dysfunction in glaucoma. Schematic representation of the molecular and cellular processes contributing to retinal ganglion cell (RGC) pathology in normotensive and high intraocular pressure (IOP) glaucoma. IOP-dependent and IOP-independent stressors impair ocular perfusion, leading to ischemia. Ischemic stress induces mitochondrial dysfunction, excitotoxic glutamate signaling, and neuroinflammatory responses, including glial activation and cytokine release. These mechanisms collectively drive progressive RGC degeneration, synaptic loss, and impaired neuroplasticity. Abbreviations: OBF, ocular blood flow; IOP, intraocular pressure; TNF-α, tumor necrosis factor α; IL, interleukin. Some elements of this illustration were adapted from images available on Freepik (https://www.freepik.com/free-photos-vectors/neuron; accessed on 29 January 2026) and NIH (National Institutes of Health) BioArt public domain (https://bioart.niaid.nih.gov; accessed on 19 January 2026).

As it has been shown that, paradoxically, acute, transient intraocular inflammation can actually activate the regenerative potential of RGCs, the major challenge is to harness the pro-regenerative aspects of inflammation while suppressing the chronic, neurotoxic inflammation that sustains RGC death in the diseased state [83] (Figure 1).

## 3. Current Neuroprotective and Neurorestorative Strategies Targeting Ischemia-Induced Neurodegeneration

Ischemic injury initiates a complex cascade of metabolic failure, excitotoxic signaling, mitochondrial dysfunction, oxidative stress, and neuroinflammation that ultimately disrupts neuronal connectivity and viability across the nervous systems. In parallel, endogenous plasticity mechanisms, including synaptic remodeling, axonal sprouting, network reorganization, and glial adaptations, are activated in an attempt to compensate for structural and functional loss. The balance between these degenerative and reparative processes critically determines neurological outcome. In glaucoma, a chronic optic neuropathy characterized by progressive RGC degeneration, vascular dysregulation and ischemia-driven plasticity failure represent central yet insufficiently targeted components of disease pathogenesis [84,85,86].

Experimental and clinical studies of cerebral ischemia have demonstrated that the adult nervous system retains a substantial, though constrained, capacity for plastic reorganization. Following stroke, perilesional cortical regions undergo changes in excitatory–inhibitory balance, dendritic spine turnover, synaptic efficacy, and functional connectivity, enabling partial recovery of lost functions [87,88,89].

Glaucoma shares key pathophysiological features with cerebral ischemia, including impaired perfusion, mitochondrial vulnerability, excitotoxic stress, and chronic inflammation [84,90]. Importantly, synaptic and dendritic degeneration within the retina and optic nerve often precede irreversible RGC death, indicating that impaired plasticity is an early and therapeutically accessible event [86,91]. These parallels support the relevance of ischemic CNS models for understanding and modulating plasticity in glaucomatous optic neuropathy.

### 3.1. Excitotoxicity, Calcium Dysregulation and Oxidative Stress

Excessive glutamatergic signaling is a hallmark of ischemic neuronal injury. Following ischemia, elevated extracellular glutamate leads to sustained activation of NMDA receptors, resulting in calcium overload, mitochondrial dysfunction, and activation of apoptotic pathways [92]. In both cerebral ischemia and glaucoma, NMDA receptor mediated excitotoxicity contributes to neuronal degeneration within vulnerable populations, including cortical pyramidal neurons and RGCs [86,93]. While early therapeutic strategies focused on blocking NMDA receptor-mediated calcium influx, emerging evidence indicates that NMDA receptors also regulate ischemic injury through non-ionotropic, metabotropic signaling pathways that influence gene expression, cytoskeletal dynamics, and plasticity-related processes [94]. Although NMDA antagonists such as MK-801 demonstrate neuroprotective effects in experimental ischemia models [92], their clinical translation has been limited by narrow therapeutic windows and unacceptable side effects [95]. In glaucoma, similar limitations have curtailed enthusiasm for direct NMDA receptor blockade, highlighting the need for approaches that preserve physiological synaptic transmission while restoring adaptive plasticity.

Calcium overload represents a convergent mechanism linking excitotoxicity, mitochondrial failure, and cell death. Calcium channel blockers (CCBs) reduce ischemia-induced calcium influx in neurons and improve vascular perfusion by promoting vasodilation [96]. In experimental models of cerebral ischemia, agents such as nimodipine reduce infarct size and preserve neuronal viability [96,97]. In ophthalmology, CCBs have attracted interest for their potential neuroprotective effects in glaucoma. Clinical and epidemiological studies suggest that long-term systemic use of CCBs may influence glaucoma risk and progression, possibly through improved OBF and reduced ischemic stress [85,97,98]. However, while CCBs may attenuate vascular and ionic components of ischemia, they do not directly engage synaptic repair or network-level plasticity mechanisms, limiting their disease-modifying potential [85].

The nervous system is particularly susceptible to oxidative damage due to high metabolic demand, abundant lipid membranes, and limited antioxidant capacity. Ischemia–reperfusion injury exacerbates ROS production, contributing to synaptic dysfunction and neuronal loss. There’s growing interest in developing new strategies, including exogenous antioxidant supplementation and ways to support the brain’s natural antioxidant systems, to help prevent or reduce CNS disorders and brain injury [99]. One limitation of antioxidant approaches is that they primarily target downstream injury pathways without restoring the plasticity programs required for long-term circuit stabilization and functional recovery. In glaucoma, oxidative stress contributes to RGC vulnerability, but antioxidant monotherapy has limited effects on disease progression [86].

### 3.2. Neurotrophic Factors and Plasticity Support

Neurotrophic signaling is essential for neuronal survival, synaptic maintenance, and adaptive plasticity following ischemic injury. BDNF and nerve growth factor (NGF) have demonstrated robust neuroprotective and plasticity-enhancing effects in models of cerebral ischemia, promoting synaptic remodeling, axonal growth, and functional recovery [100,101,102]. Blockade of BDNF signaling markedly impairs post-ischemic recovery, underscoring its central role in neural repair [100].

In glaucoma, impaired retrograde transport of neurotrophic factors along the optic nerve contributes to RGC degeneration [86,91]. Although exogenous delivery of neurotrophic factors shows promise, translation has been limited by short half-lives, delivery challenges, and the need for sustained signaling [31,90]. These limitations have driven interest in pharmacological strategies capable of activating endogenous neurotrophic pathways rather than relying on protein replacement.

### 3.3. Structural Constraints on Plasticity and Cell-Based Neurorestorative Strategies

Plasticity in the adult CNS is constrained by a range of structural and molecular mechanisms that stabilize mature neural circuits but limit regenerative capacity following injury. Contributors include inhibitory extracellular matrix components, such as perineuronal nets and myelin-associated inhibitors, which restrict axonal sprouting and synaptic remodeling under physiological conditions. After ischemic injury, partial degradation of these structures occurs in perilesional regions, coinciding with transient increase in plastic potential [88,89]. Experimental manipulation of extracellular matrix components, such as enzymatic degradation of chondroitin sulfate proteoglycans, enhances axonal sprouting and synaptic density in models of ischemic injury [88]. However, these approaches are invasive, difficult to control temporally and spatially, and difficult to translate to chronic neurodegenerative conditions such as glaucoma, where sustained and precisely regulated modulation of plasticity must be achieved safely and selectively in order to preserve visual function.

Stem cell-based therapies have been explored as a multitarget strategy for ischemia-associated neurodegeneration, because of their combined neuroprotective, immunomodulatory, and pro-angiogenic properties [103]. In chronic ocular conditions such as glaucoma, preclinical studies using mesenchymal stem cells (MSCs) and their secreted products indicate that these cells can create a supportive microenvironment that limits RGC loss and attenuates inflammation and oxidative stress, even in the absence of direct neuronal replacement [104,105,106]. Increasing evidence suggests that the therapeutic effects of stem cells are predominantly mediated through paracrine mechanisms, including the release of growth factors, cytokines, and extracellular vesicles, rather than stable functional integration into existing neural circuits [107]. Consistent with this view, stem cell-derived extracellular vesicles, including exosomes, have gained attention as a cell-free alternative capable of recapitulating many of the beneficial paracrine effects of stem cell therapy [108,109,110]. Exosomes derived from MSCs have been shown to reduce RGC apoptosis, preserve retinal structure, and promote angiogenic and neuroprotective signaling in experimental models of chronic ocular hypertension and retinal ischemia, partly through the transfer of bioactive microRNAs that regulate survival and plasticity-related pathways [106,108,111,112].

Despite these encouraging findings, outcomes of stem cell-based and exosome-mediated therapies have been variable, and clinical translation remains challenging. Reported limitations include inconsistent efficacy, potential adverse tissue responses, uncertainties regarding optimal cell or vesicle sources, delivery routes, dosing regimens, and unresolved concerns related to long-term safety in the context of slowly progressive glaucomatous degeneration [113,114,115,116]. Collectively, while stem cell-based and extracellular vesicle-based strategies provide important mechanistic insights and demonstrate proof-of-concept for modulating ischemia-driven neurodegeneration, their current limitations highlight the need for complementary therapeutic approaches capable of safely and systemically enhancing neuroplasticity, dampening neuroinflammation, and improving metabolic resilience in chronic diseases such as glaucoma [52,111,112,117].

### 3.4. Implications for Glaucoma Therapy

Despite extensive research, most neuroprotective and neurorestorative strategies for ischemic injury have failed to translate into effective long-term therapies, and their translation to glaucoma presents unique challenges related to disease chronicity, compartmentalized anatomy, and the progressive nature of RGC loss. Glaucoma, as a chronic ischemic neurodegenerative disease, requires interventions capable of restoring synaptic integrity, metabolic resilience, and circuit-level plasticity over prolonged periods [84,89,91].

These limitations provide the conceptual framework for the emergence of plasticity-centered pharmacological strategies, including psychoplastogens. By simultaneously engaging neurotrophic signaling, synaptic remodeling, mitochondrial support, and anti-inflammatory pathways, such agents may overcome the shortcomings of traditional neuroprotective approaches and offer a more integrated therapeutic strategy for glaucomatous neurodegeneration.

## 4. Psychoplastogens: Mechanisms and Therapeutic Promise

**Figure 2:**
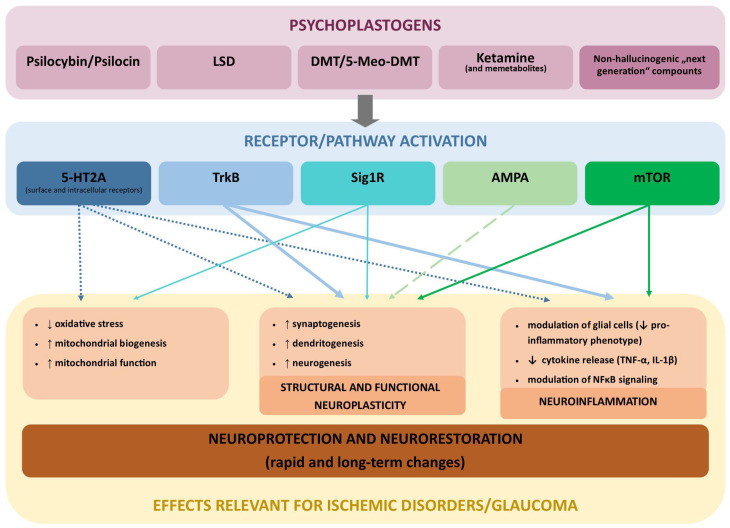
Neuroprotective and neurorestorative mechanisms of psychoplastogens in glaucoma. Schematic overview of the rapid and long-term multifaceted actions of psychoplastogens relevant for glaucoma. Through modulation of neuroplasticity-related signaling pathways, regulation of neuroinflammatory responses, and enhancement of metabolic resilience, psychoplastogens may promote retinal ganglion cell survival, functional recovery, and regenerative capacity. Abbreviations: LSD, lysergic acid diethylamide; DMT, N,N-dimethyltryptamine; 5-Meo-DMT, 5-methoxy-dimethyltryptamine; TrkB, tyrosine receptor kinase B; Sig1R, sigma-1 receptor; AMPA, α-amino-3-hydroxy-5-methyl-4-isoxazolepropionic acid; mTOR, mammalian target of rapamycin.

Psychoplastogens are expanding class of small molecules capable of inducing rapid, robust, and sustained neuroplastic changes in the CNS. Importantly for glaucoma, many of the molecular targets and signaling pathways engaged by psychoplastogens are not restricted to cortical circuits but are also expressed in the retina, optic nerve, and ocular outflow tissues. RGCs, retinal glia, and vascular elements express key psychoplastogen-responsive targets, including serotonin receptors, sigma-1 receptors (Sig1R), and downstream plasticity-associated signaling pathways such as BDNF–TrkB, mTOR, and AMPA receptor pathways [118,119,120,121]. These systems are directly implicated in the regulation of neuronal survival, metabolic resilience, inflammation, and synaptic integrity, processes that are disrupted early in glaucomatous neurodegeneration (Figure 2).

Beyond their established effects in psychiatric disease, accumulating evidence from CNS ischemia models demonstrates that psychoplastogens exert potent neuroprotective and anti-inflammatory under conditions of hypoxia, oxidative stress, and excitotoxic injury [122,123,124,125,126]. These pathophysiological mechanisms closely mirror those observed in glaucoma, where chronic microvascular compromise, mitochondrial dysfunction, glutamate dysregulation, and sustained neuroinflammation contribute to progressive RGC loss and optic nerve degeneration [11,12,18,26,127]. Moreover, serotonergic signaling within ocular tissues influences aqueous humor dynamics and OBF, suggesting that certain psychoplastogens may simultaneously modulate neurodegeneration and IOP, the primary modifiable risk factor for glaucoma [128,129,130].

Unlike traditional neurotrophic or neurorestorative therapies, psychoplastogens promote structural and functional plasticity after single or intermittent dosing [34,131,132,133], offering a new opportunity in the treatment of psychiatric, neurodegenerative, and ischemic disorders. Their ability to engage many different intracellular pathways [134,135,136,137,138,139], positions them as uniquely suited candidates for addressing the multifactorial and ischemia-driven nature of glaucomatous pathology. In this section, we first outline the defining features and mechanistic basis of psychoplastogens, followed by a focused discussion of their relevance to ischemic injury, retinal neuroprotection, and optic nerve degeneration.

### 4.1. Molecular and Cellular Mechanisms of Psychoplastogen Action

#### 4.1.1. Definition and Criteria of Psychoplastogens

The term “psychoplastogen” was introduced to describe molecules that rapidly and sustainably enhance neuronal structural and functional plasticity following brief exposure [34]. This class encompasses classical psychedelics, such as lysergic acid diethylamide (LSD), psilocybin, mescaline, DMT, and psychoactive agents like dissociative anesthetic ketamine and its metabolites, 3,4-methylenedioxymethamphetamine (MDMA) and deliriants [140,141]. Classical psychedelics are prototypical psychoplastogens, but significant recent efforts have focused on engineering safer, non-hallucinogenic derivatives with similar molecular effects but improved therapeutic indices [131,142]. Examples are tabernanthalog (TBG), DM506, (+)-JRT and DLX-001, engineered to retain the desired plasticity-promoting effects while eliminating psychotropic liability [38,143,144,145,146,147].

Unlike chronic antidepressants or trophic factors, psychoplastogens induce rapid dendritic spinogenesis, synaptic strengthening, and circuit-level remodeling [132,133,148]. These effects typically emerge within hours and persist for weeks, enabling durable therapeutic outcomes even after single administrations [131,149]. Recent research has revealed broad neurorestorative and anti-inflammatory properties, positioning psychoplastogens as multitarget therapeutics with relevance to ischemic and neurodegenerative conditions, including glaucoma [126,150,151,152,153].

#### 4.1.2. Receptor-Level Mechanisms Initiating Psychoplastogenic Signaling

For most classical psychedelics, activation of the 5-HT2AR is a central initiating step [134,148]. Early assumptions linked psychedelic effects exclusively to surface 5-HT2AR activation, but recent studies demonstrate that intracellular 5-HT2AR pools are crucial for plasticity induction [154]. This distinction is critical because it explains why endogenous serotonin, despite its high affinity, does not typically trigger plasticity observed with psychedelics. Activation of intracellular 5-HT2ARs increases BDNF release and facilitates AMPA receptor trafficking, suggesting a connection between serotonergic and glutamatergic plasticity mechanisms [132,142,146]. Psilocybin and psilocin robustly activate 5-HT2ARs in cortical pyramidal neurons, driving rapid spinogenesis and functional synaptic strengthening [132,133]. These cellular changes correspond with enhanced prefrontal connectivity and extended behavioral improvements.

One of the major downstream mechanism involves the TrkB receptor, the high-affinity receptor for BDNF [34,138,149], essential for neuronal survival, synaptic and dendritic growth, and maturation [155]. Recent evidence indicates that several classical psychedelics promote plasticity by directly binding to the TrkB receptor and acting as positive allosteric modulators of BDNF-TrkB signaling [138], thus facilitating receptor activation, and promoting downstream signaling. Direct TrkB binding provides an explanation for why psychedelics potentiate BDNF-dependent plasticity and may contribute to their long-lasting synaptogenic effects.

Another important pathway involves activation of mTORC1. mTORC1 regulates protein synthesis necessary for synapse formation and dendritic growth, and its activation is observed following exposure to both hallucinogenic and non-hallucinogenic psychoplastogens [34,142,145,146,150,156].

Sustained AMPA receptor throughput is essential for both the initiation and the maintenance of psychoplastogen-induced neuronal growth, and the inhibition of AMPA receptors has been shown to block the entire growth phenotype induced by psychoplastogens [142,146].

The Sig1R is another significant mechanism relevant to psychoplastogens. Sig1R is expressed in neurons, astrocytes, and microglia, where it regulates neurite outgrowth, synaptogenesis, myelination, and regenerative signaling [157]. Its activation stabilizes ER–mitochondrial interactions, improves calcium buffering, enhances mitochondrial ATP production, protects against oxidative stress, and reduces expression of pro-apoptocic genes [158,159]. DMT is a potent Sig1R agonist and exerts anti-inflammatory, anti-apoptotic, and cytoprotective actions in models of ischemia and hypoxia [124,160]. These anti-ischemic effects directly overlap with mechanisms relevant to glaucomatous optic neuropathy.

#### 4.1.3. Mitochondrial Modulation and Metabolic Support

Ischemic insults, including those implicated in glaucoma, also induce mitochondrial dysfunction, ATP depletion, and ROS generation [126,127], contributing to RGC vulnerability in glaucoma. By activating 5-HT2AR, psychoplastogens can mediate mitochondrial biogenesis and/or increase mitochondria oxidative capacity, enabling rapid growth and synaptic maintenance even under metabolic stress [150,161,162,163]. DMT via Sig1R activation promotes mitochondrial stabilization, reduces ROS production, and enhances ATP availability [122,125,160]. Ketamine and its metabolites enhance synaptic energy metabolism and reduce inflammation-driven mitochondrial dysfunction [164]. Given the high metabolic demands of RGCs, mitochondrial support is especially relevant for ischemia-induced damage. These effects are a key factor distinguishing psychoplastogens from purely synaptic modulators.

#### 4.1.4. Immunomodulatory and Anti-Inflammatory Effects

Neuroinflammation, characterized by chronic overactivation of microglia and the release of cytotoxic molecules, is a critical component of neurodegenerative and neuropsychiatric disorders [151,153,165]. Psychedelics display potent anti-inflammatory properties by binding to 5-HT2A expressed on immune cells [137,152,153,166,167]. Psilocin, active metabolite of psilocybin, modulates neuroimmune functions of microglia in a 5-HT2 receptor-dependent manner [139,151]. Study from Wiens et al. [151] showed that psilocin significantly inhibits the release of cytotoxic agents, specifically ROS and NO, which contribute to oxidative damage in neuropathologies. This inhibitory activity is observed even under stimulation by various inflammatory mediators, suggesting a broad anti-neuroinflammatory potential. Similarly, the psychedelic 5-HT2A agonist 2,5-dimethoxy-4-iodoamphetamine (R)-DOI) produces potent anti-inflammatory effects in peripheral tissues by suppressing TNF-α-induced inflammation at remarkably low (picomolar) concentrations, suggesting functional selectivity independent of overall receptor density [152,168,169]. The anti-inflammatory actions of ketamine are theorized to involve the NFκB cascade, demonstrating its role in regulating neuroplasticity, neurogenesis, and neuronal survival alongside neuroinflammation and cell death [170]. Serotonergic 5-HT2AR agonists similarly suppress inflammatory signaling by modulating the NFκB, mTOR, and phosphatidylinositol-3-kinase/protein kinase B (PI3K/Akt) pathways [153]. Immunomodulatory and anti-inflammatory effects of psychedelics are discussed in detail in de Deus et al. [153], Low et al. [166], and Inserra et al. [137].

Treatment with non-hallucinogen psychoplastogen TBG was shown to normalize microglial density and morphology and reverse cognitive deficits associated with elevated microglial activation in a cancer-related cognitive impairment mouse model [171]. In vitro, TBG partially suppressed neuroinflammatory gene expression in BV-2 microglial cells exposed to tumor-derived conditioned medium.

Such immunomodulatory actions could help counteract the glial reactivity and inflammatory signaling seen in glaucoma.

#### 4.1.5. Structural and Functional Plasticity Induced by Psychoplastogens

Classical psychedelics and non-hallucinogenic analogs promote both structural and functional neuroplasticity. Psychedelics increase dendritic spine density, dendritic complexity, and synaptic bouton number in cortical neurons within 24 h [132,133]. These effects persist for weeks and correlate with improved cognitive performance and mood-related behaviors in animals. Functional changes include increased AMPA/NMDA ratio, enhanced LTP-like synaptic potentiation, strengthening of frontal cortical connectivity, rewiring of stress-impaired circuits [140,172]. These durable changes support the concept that psychoplastogens restore lost or compromised neural connectivity, an appealing mechanism for glaucomatous neurodegeneration, where dendritic pruning and synaptic loss precede RGC death.

#### 4.1.6. Immediate Early Gene Activation and Glutamate Burst

While the 5- HT2AR/TrkB/mTOR/AMPA cascade is essential for neuroplasticity, key differences exist between hallucinogenic and non-hallucinogenic psychoplastogens regarding their acute signaling profile. Hallucinogenic psychedelics reliably induce two immediate events: glutamate burst, a rapid increase in glutamate release in the cortex, and immediate early gene (IEG) activation, a rapid induction of plasticity-related genes within the cortex [142,173]. In contrast, studies confirm that the non-hallucinogenic analog TBG, despite promoting cortical neuron growth and long-lasting antidepressant effects comparably to 5-methoxy-N,N-dimethyltryptamine (5-MeO-DMT), does not induce an immediate glutamate burst or proximate IEG activation. This crucial finding suggests that the glutamate burst and prompt IEG activation are tightly linked to the acute psychedelic experience but are not required for the subsequent sustained neuroplastic changes [142]. This decoupling mechanism supports the development of non-hallucinogenic compounds (“neuroplastogens”) that are highly effective without subjective effects.

### 4.2. Evidence from CNS Ischemia and Retinal and Optic Nerve Research

#### 4.2.1. Psychoplastogen-Induced Neuroprotection and Plasticity in CNS Ischemia

The pathophysiology of glaucoma includes chronic vascular dysregulation and reduced microcirculatory perfusion, leading to ischemic and hypoxic stress within the retina and optic nerve head [18,26,27]. These insults trigger excitotoxicity, mitochondrial failure, oxidative stress, neuroinflammation, and ultimately progressive RGC degeneration, pathways that closely mirror those observed in cerebral ischemia and stroke [122,123,124,125,126]. Consequently, preclinical and clinical evidence from CNS ischemia models provides relevant framework for evaluating the neuroprotective and neurorestorative potential of psychoplastogens in glaucomatous neurodegeneration.

The psychoplastogen DMT and its analogs, demonstrate neuroprotective and immunomodulatory effects across multiple ischemia-relevant models, dominantly through Sig1R activation. Data from in vitro models demonstrates that DMT significantly increases the survival of human primary iPSC-derived cortical neurons and microglia-like immune cells when exposed to severe hypoxic conditions [124]. This cytoprotective effect under hypoxia is primarily mediated by Sig1R activation. DMT was also suggested to have anti-inflammatory effects mediated by Sig1R activation [174]. Namely, it reduced production of pro-inflammatory cytokines (IL-1β, IL-6, TNF-α, IL-8), increased the anti-inflammatory cytokine IL-10, and suppressed immune responses. In human cerebral organoids, tryptamine 5-MeO-DMT favorably alter the cerebral proteome, upregulating factors involved in long-term potentiation and dendritic spine maturation while inhibiting factors related to neurodegeneration [159]. In murine models, DMT limited infarct size in a rodent model of focal cerebral ischemia, and facilitated the recovery of motor function following ischemic injury [123]. Szabó et al. [125] demonstrated that DMT protects astrocytes against ischemic injury, and suppresses ischemia/reperfusion-related apoptosis in the nervous tissue of the rat. In middle cerebral artery occlusion model, DMT significantly reduced the volume of infarct lesions and associated cerebral edema, restored BBB integrity, attenuated astrocyte dysfunction, suppressed microglial activation, shifted the systemic immune response toward an anti-inflammatory state by suppressing pro-inflammatory cytokines (TNF−α,IL−1β,IL−6) and chemokines while increasing anti-inflammatory IL-10 and neuroprotective BDNF [122]. Collectively, these findings support dominantly Sig1R-mediated mechanism by which DMT and possibly related psychoplastogens promote neuroprotection, immune regulation, and structural plasticity in ischemia-vulnerable neural tissue.

Classical serotonergic psychedelics also show compelling neuroprotective effects in ischemic conditions. Psilocybin and its active metabolite psilocin have been shown to reduce glutamate-mediated excitotoxicity in neuronal cultures exposed to oxygen–glucose deprivation and to significantly decrease infarct size and neurological deficits in rat stroke models [126]. The same study founded increase in the expression of neuronal and synaptic markers, decreased activation of microglia and upregulated BDNF expression.

Although ketamine is not a classical psychedelic, it promotes both structural and functional neuroplasticity through NMDA receptor-independent mechanisms, engaging AMPA receptor activation and downstream mTORC1 signaling, sharing this as a convergent mechanism with classical psychedelics [149,175,176]. Preclinical research suggests that (R)-ketamine, but not (S)-ketamine, can ameliorate neuronal brain injury when administered after a cerebral ischemic stroke [177]. Preclinical and clinical data suggest ketamine metabolites such as (2R,6R)-hydroxynorketamine promote post-ischemic synaptogenesis, restore dendritic architecture, and attenuate microglial activation independently of NMDA receptor antagonism [175,176,178,179].

These findings underscore the relevance of psychoplastogens in restoring plasticity within ischemia-compromised neural circuits rather than merely preventing acute cell death. However, the therapeutic potential of psychoplasatogens in ischemia is supported predominantly by preclinical evidence, with limited clinical trial data available, highlighting the challenges of translating findings from animal models to humans and the need for further studies to establish efficacy and safety in ischemia-related diseases [180].

#### 4.2.2. Ocular Relevance of Psychoplastogen-Responsive Pathways in the Retina and Optic Nerve

Potential for translational application of psychoplastogens to glaucomatous ischemic neurodegeneration is driven by the fact that their molecular targets are expressed and functionally active in both retinal and optic nerve tissues. Multiple convergent signaling pathways engaged by psychoplastogens, including Sig1R, serotonergic receptors, BDNF-TrkB signaling, mTOR, and AMPA receptor-dependent mechanisms, have been directly implicated in retinal physiology, stress responses, and neurodegeneration.

Sig1Rs are widely expressed in RGCs, photoreceptors, interneurons, Müller glia, and optic nerve astrocytes, positioning Sig1R as a central regulator of retinal stress resilience [181,182]. Studies demonstrate that Sig1R activation mitigates oxidative stress, attenuates endoplasmic reticulum stress, stabilizes mitochondrial function, and suppresses apoptotic signaling in models of retinal ischemia, excitotoxicity, optic nerve injury, and glaucoma [119,181,182,183]. Sig1R agonists preserve RGC survival, maintain retinal structure, and reduce neuroinflammatory signaling, highlighting Sig1R as a validated neuroprotective target in ocular neurodegeneration. In glaucoma rat models, Sig1R activation by agonists like pridopidine or (+)-pentazocine provides significant neuroprotection to RGC somas and axons [184,185]. A key contributor to RGC death in glaucoma is mitochondrial dysfunction. Geva et al. [184] demonstrated that Sig1R agonists pridopidine rescue mitochondrial membrane potential and enhance basal and maximal respiration, as well as ATP production in stressed neurons. Glaucoma also involves the obstruction of axonal transport of BDNF, which is vital for RGC health and neuroprotection [186,187]. Sig1R agonists upregulate the secretion and transport of BDNF from neurons and astrocytes, promoting neuronal differentiation and survival [184,186]. Glaucoma patients often exhibit high levels of ROS [184,187]. Sig1R modulates nuclear factor erythroid 2-related factor 2, a master regulator of antioxidant response element-dependent genes, to enhance the production of antioxidant proteins and neutralize ROS [119,180,183]. NMDA receptor is considered the main mediator of glutamate excitotoxicity damage in the retina, resulting in toxic calcium influx and RGC death, and consequently involved in glaucoma, ischemic and diabetic retinopathy, and optic neuritis [121,187]. Sig1R modulate the function of several ligand-gated and voltage-gated ion channels, including NMDA-and AMPA-mediated cation channels, and calcium channels [183]. Sig1R agonists associate with and inhibit L-type voltage-gated calcium channels and thus modulate NMDA receptor activity to maintain calcium homeostasis [186]. Also, it has been demonstrated that Sig1R activation effectively attenuates the expression of various pro-apoptotic markers, thereby preventing programmed cell death in the retina [181]. Glaucoma involves the reactive gliosis of Müller cells, microglia, and astrocytes [121,183]. Sig1R agonists were found to decrease the release of proinflammatory cytokines and chemokines by Müller cells and microglia [183,188,189].

Serotonergic psychoplastogens offer a unique advantage by targeting both neurodegeneration and the main risk factor, IOP. Multiple serotonin receptors and transporters, including 5-HT1A, 5-HT2 family receptors, and the serotonin transporter, are expressed in the retina and optic nerve, where they modulate synaptic transmission, vascular tone, and metabolic activity [190,191,192,193]. The 5-HT2AR is highly expressed in ocular outflow tissues, including the human ciliary body, ciliary epithelium, and trabecular meshwork [129]. Activation of 5-HT2ARs stimulates phosphoinositide hydrolysis and mobilizes intracellular calcium in human ciliary muscle and human TM cells [194]. This signaling cascade results in the production and secretion of matrix metalloproteinases, which remodel the extracellular matrix, enhancing aqueous humor outflow, thus significantly lowering IOP [128,129,130]. Clinically relevant 5-HT2 agonists, such as AL-34662, have demonstrated significant IOP reduction in ocular hypertensive Cynomolgus monkeys, considered a reliable model for humans [129]. Activation of retinal 5-HT1A receptors has been shown to modulate presynaptic neurotransmission, enhancing GABAergic input and reducing excitotoxic glutamatergic signaling, which could increase RGC viability in chronic glaucoma models [190]. Also, 5-HT1A receptor activation has been shown to influence retinal blood flow and IOP, processes directly implicated in glaucoma pathophysiology [195,196]. Taken together, the expression and functional roles of serotonergic receptors in retinal neurons, vascular elements, and aqueous humor outflow pathways suggest that serotonergic psychoplastogens may offer a uniquely integrated therapeutic approach in glaucoma, with the potential to simultaneously modulate IOP, ischemia-related stress, and RGC vulnerability through neuromodulatory and plasticity-promoting mechanisms.

BDNF is considered the most critical neurotrophin for the survival and differentiation of RGCs, acting as a natural defense mechanism against the neurodegeneration seen in glaucoma [100,101,102,187,197]. Impaired retrograde transport of BDNF has been implicated in glaucomatous degeneration [86,91,198]. The TrkB receptor, which mediates the effects of BDNF, often becomes downregulated or desensitized in the retina under conditions of chronic glaucomatous stress, limiting the cell’s ability to respond to surviving neurotrophic signals [121]. Although exogenous delivery of neurotrophic factors has shown promise in glaucoma treatment, translation has been limited by short half-lives, delivery challenges, the requirement for sustained signaling to maintain therapeutic efficacy [31,90,197]. Augmentation of TrkB signaling through gene therapy or pharmacological approaches, including small-molecule TrkB agonists, enhances RGC survival and axonal integrity in experimental glaucoma, underscoring the therapeutic relevance of BDNF signaling pathways [187,197]. In this context, psychoplastogens represent a complementary therapeutic opportunity, as several compounds in this class have been shown to engage TrkB-dependent signaling indirectly or allosterically, while simultaneously modulating upstream serotonergic, metabolic, and inflammatory pathways. Rather than acting as direct neurotrophic substitutes, psychoplastogens may facilitate endogenous plasticity and trophic support through transient but coordinated activation of convergent survival and remodeling pathways. This multimodal mechanism could help overcome some of the limitations associated with isolated neurotrophin delivery, although direct evidence in chronic ocular disease models remains limited and warrants further investigation.

The mTOR and AMPA receptor-dependent plasticity pathways, while less extensively characterized in ocular tissues than in cortical circuits, are increasingly recognized as contributors to retinal metabolic adaptation and synaptic remodeling following injury [155,199,200]. The mTOR pathway plays an important role in retinal and optic nerve health and disease, regulating metabolism, growth, inflammation, and neuronal survival [155]. Dysregulated mTOR activity has been implicated in glaucoma, diabetic retinopathy, and optic nerve injury, and experimental modulation of mTOR influences retinal cell survival and structural outcomes [199]. However, data considering mTOR pathway manipulations suggest that therapeutic interventions should be precisely balanced; while mTOR-mediated autophagy can act as a survival mechanism, its over-regulation or excessive inhibition via rapamycin has also been linked to increased RGC apoptosis in certain glaucomatous contexts [155]. According to Zhu et al. [201], regulation of mTOR activity varies among RGC types with differential susceptibility to ischemia/reperfusion injury.

Collectively, these findings indicate that the key molecular targets engaged by psychoplastogens are endogenously expressed and functionally active in the retina, optic nerve head, and optic nerve. Their roles in mitochondrial homeostasis, neuroinflammation, synaptic plasticity, vascular regulation, and IOP control provide biological rationale for cautiously exploring psychoplastogen-based approaches to the neurodegenerative and vascular components of glaucoma and related ischemia-associated ocular disorders.

## 5. Integration of Psychoplastogen-Based Therapies with Existing Therapies, Challenges, and Future Directions

Glaucoma treatment has historically centered on reducing IOP, the only proven modifiable risk factor for disease progression. Although IOP-lowering strategies, including prostaglandin analogs, β-blockers, carbonic anhydrase inhibitors, Rho kinase inhibitors, selective laser trabeculoplasty, and surgical approaches, remain indispensable, they do not sufficiently address the ischemic, inflammatory, and neurodegenerative processes that compromise RGC survival [4,31,127,202]. As understanding of glaucoma as a chronic neurodegenerative disorder deepens, integrating psychoplastogen-based neuroprotection with existing therapeutic approaches is emerging as a promising direction.

### 5.1. Complementarity with Current Glaucoma Therapies

Modern glaucoma management is moving toward multimodal approaches that combine IOP control with neuroprotection, vascular stabilization, and anti-inflammatory interventions. Neurotrophic factors such as BDNF, NGF, and related pathways have shown potential to enhance RGC resilience but face challenges regarding delivery and duration of effect [31,90].

In this context, psychoplastogens, substances capable of rapidly inducing structural and functional neural plasticity, offer mechanisms distinct from classical ocular therapeutics. Their engagement of BDNF–TrkB signaling, Sig1R activity, and intracellular 5-HT2AR pathways aligns well with known deficits in glaucomatous neurodegeneration, including synaptic loss, mitochondrial dysfunction, and impaired axonal transport. This provides a powerful, small-molecule mechanism for RGC survival, potentially overcoming the poor pharmacokinetics and complex delivery requirements associated with protein-based neurotrophic factors [119,127].

Ischemia and reperfusion injury contribute significantly to glaucomatous pathology through oxidative damage, microglial activation, and mitochondrial compromise [25,30]. Psychoplastogens offer mechanistic advantages in these contexts. Sig1R activation by DMT and derivatives stabilizes mitochondrial function, reduces oxidative stress, and protects against hypoxic injury [119,160]. BDNF–TrkB pathway engagement promotes dendritic spine recovery and synaptic remodeling, processes impaired early in glaucoma [33,138]. Potent immunomodulatory effects have been demonstrated for various psychoplastogens. Ketamine and its metabolites capacity for synaptogenesis, immunomodulation, and mGlu2-mediated signaling [164,175] directly addresses mechanisms implicated in RGC degeneration, such as inflammation, excitotoxicity, and metabolic stress [27,203].

These mechanisms mirror emerging neuroprotective strategies in glaucoma that aim to counteract oxidative stress, restore axonal transport, and modulate microglial activation [18,27].

### 5.2. Delivery Routes and Ocular Pharmacokinetics

A critical translational barrier for psychoplastogens in glaucoma is achieving therapeutic concentrations in the posterior segment, particularly in the retina and optic nerve. The eye is protected by a series of static and dynamic anatomical and physiological barriers that limit drug penetration and residence time, including the tear film, cornea, conjunctiva, sclera, blood–aqueous barrier, and the blood–retina barrier, which comprises tight junctions in retinal capillary endothelia and retinal pigment epithelium [204,205]. These barriers evolved to preserve ocular homeostasis but also restrict access of systemically administered or topically applied agents to the posterior segment, causing low drug bioavailability [206,207]. The tear film and nasolacrimal drainage quickly dilute and clear topical formulations, resulting in a very small portion of drug reaching deeper ocular tissues, and even less reaching the retina [204,205].

While systemic delivery of psychoplastogens such as DMT, ketamine, or psilocybin may produce neuroprotective effects and promote plasticity in CNS models, systemic routes are limited by the blood–retina barrier and efflux transporters, low posterior bioavailability, and potential systemic side effects including psychoactivity and cardiotoxicity, which are not desirable for chronic ophthalmic indications. Recent glaucoma-focused drug delivery reviews strongly emphasize that local delivery to the vitreous or periocular space is often necessary to overcome these barriers and achieve sustained posterior segment concentrations [127,204,205]. Intravitreal injection remains the gold standard for posterior segment targeting, capable of circumventing the blood–retina barrier and delivering drugs directly into the vitreous cavity, but it is invasive and can be associated with risks such as endophthalmitis, hemorrhage, and retinal detachment with repeated administration [205,208].

To minimize invasive procedures while improving posterior bioavailability, multiple alternative routes and advanced formulations are under investigation. Subconjunctival, transscleral, and suprachoroidal routes offer semi-invasive access to periocular tissues adjacent to the retina, though diffusion across multiple tissue layers remains a significant challenge [127,204]. Transport across the inner limiting membrane and vitreous gel also impedes drug penetration to the inner retina, particularly for larger or hydrophilic molecules [204,209].

Advanced controlled-release and nanotechnology-based delivery systems are increasingly explored to address these limitations. Sustained-release implants and biodegradable polymer systems can prolong drug residence time while reducing the frequency of intravitreal injections [127]. Nanocarriers, including nanomicelles, dendrimers, and lipid-based nanoparticles, enhance ocular bioavailability by facilitating diffusion across ocular barriers, protecting payloads from enzymatic degradation, and enabling targeted delivery to retinal tissues, with preclinical models showing improved retinal retention and extended pharmacokinetics (e.g., enhanced intravitreal half-life and reduced clearance) [205,210]. For example, nanocarriers encapsulating neuroprotective agents such as dehydroepiandrosterone have demonstrated extended preservation of RGCs in experimental models, suggesting proof of concept for sustained posterior segment delivery with reduced dosing frequency [127].

Despite these advancements, there remains a lack of detailed pharmacokinetic data specific to psychoplastogens in ocular tissues, including routes of absorption, distribution, metabolism, and elimination within the retina and optic nerve. Future research needs to quantify retinal bioavailability, barrier penetration, half-life in vitreous and retinal tissues, and safety profiles associated with repeated or sustained administration of both classic and non-hallucinogenic psychoplastogens. Such studies will be essential to refine delivery strategies that maximize therapeutic potential while minimizing safety risks, particularly for chronic diseases such as glaucoma.

### 5.3. Safety and Hallucinogenic Liability: Prospects of Non-Hallucinogenic Analogs

The psychoactive properties of classical psychedelics present a major clinical hurdle, requiring extensive supervision and restricting access, especially for patients with a family history of psychosis or schizophrenia [144,211]. Non-hallucinogenic psychoplastogens resolve this issue by decoupling therapeutic effects from subjective experience. Compounds like TBG, DM506 and (+)-JRT promote plasticity and rapid antidepressant-like effects without inducing the head-twitch response, the behavioral proxy for hallucination in rodents [131,144,212]. The lack of proximate IEG induction and glutamate burst in TBG correlates with its non-hallucinogenic profile, making analogues like TBG, DM506, and (+)-JRT prime candidates for scalable, chronic neurorestorative therapies [142,143,144,212].

Classical psychedelics and related compounds carry significant cardiotoxic risk, primarily through the potential to induce cardiac valvulopathy via chronic 5-HT2B receptor activation or cardiac arrhythmias via hERG channel inhibition [131,141,150,213]. Classical chronic use of compounds that activate the 5-HT2B receptor carries a significant risk of cardiac valvulopathy, necessitating the development of analogs highly selective for the 5-HT2A receptor and antagonistic at 5-HT2B, which is one of the goal of development of new generation of psychoplastogens. TBG and DM506 exhibit a superior safety profile over its parent compound, ibogaine, specifically by demonstrating significantly lower affinity for the hERG potassium channel, thereby mitigating the risk of cardiac arrhythmias [131,212].

Nonhallucinogenic psychoplastogens offer several transformative advantages for mental health care: by eliminating hallucinogenic liability, these compounds could be self-administered at home, without the need for in-clinic observation after administration; analogs like TBG and DM506 lack the cardiotoxicity associated with ibogaine; they can be used in population with psychedelic drug contraindication, such as those with schizophrenia or a family history of psychosis. On the other hand, they seem to have beneficial therapeutic features to the same extend as hallucinogenic psychoplastogens: they act as disease-modifying agents that promote neurorestoration by rapidly restoring atrophied dendritic branches and synapses [131,144,145]; they suppress neuroinflammation through the stabilization and normalization of microglial morphology and the activation of the Sig1R [171]; they stimulating BDNF-TrkB-mTOR neurotrophic signaling cascades [142,146]. While preclinical evidence for these compounds is promising, future human clinical trials are necessary to confirm whether these neurobiological effects translate into clinical benefits in the absence of a subjective psychedelic experience.

### 5.4. Integration with Future Clinical Frameworks

The strong conceptual link between retinal and cortical neuroplasticity underscores the broader potential of psychoplastogens. Evidence of cortical reorganization in glaucoma [33] suggests that therapies enhancing system-wide plasticity may support both retinal and central rewiring, contributing to functional recovery.

The transient nature of psychedelic effects demands accurate, translational biomarkers beyond traditional behavioral scales. Peripheral BDNF measurements are widely used as a proxy for neuroplasticity in human studies [214]. However, comprehensive meta-analyses show that peripheral BDNF levels are unreliable and do not significantly increase following acute psychoplastogen administration in humans, suggesting they are unsuitable for measuring rapid changes in neural plasticity.

Future research must prioritize objective, quantitative measures, such as positron emission tomography (PET) imaging to measure the density of synaptic markers like synaptic vesicle glycoprotein 2A [140,150], advanced neuroimaging techniques like functional magnetic resonance (fMRI) to assess functional connectivity changes [141], stimulation-based methods involving electrophysiology that assess neuroplasticity in humans [214], and multimodal approaches [36], thus providing a more informative index than static structural changes alone.

Collectively, psychoplastogens represent a mechanistically innovative class capable of addressing both upstream ischemic injury and downstream neurodegeneration. If safety and delivery challenges are resolved, these compounds could meaningfully broaden the therapeutic landscape for glaucoma and other optic neuropathies.

## 6. Conclusions

Glaucoma is increasingly recognized as a progressive neurodegenerative disorder driven not only by elevated IOP but also by ischemia, mitochondrial dysfunction, oxidative stress, and chronic neuroinflammation. These mechanisms converge to impair axonal transport, destabilize synapses, and ultimately lead to RGC death. Despite advancements in pressure-lowering therapies, a substantial proportion of patients continue to experience disease progression, highlighting the critical need for complementary neuroprotective strategies.

Psychoplastogens, compounds capable of inducing rapid and sustained neural plasticity, offer a compelling therapeutic avenue with the potential to address several key pathological features of glaucomatous degeneration. These agents act through mechanisms relevant to optic nerve injury, including BDNF–TrkB activation, Sig1R-mediated mitochondrial protection, anti-inflammatory modulation, and promotion of dendritic and synaptic remodeling. Importantly, early glaucomatous damage is characterized by synaptic and dendritic decline, suggesting that enhancing plasticity and structural resilience may be particularly impactful.

Future translational progress will depend on optimizing delivery routes, ensuring safety, and designing non-hallucinogenic analogs suitable for ophthalmic use. Combining psychoplastogens with established IOP-lowering therapies and emerging neuroprotective agents may allow for a more comprehensive strategy to preserve vision, especially in patients with vascular dysregulation or progressive disease despite controlled IOP.

Overall, psychoplastogen-based interventions represent a promising frontier in glaucoma therapeutics, as well as other ischemia-related conditions. Their multimodal mechanisms addressing ischemia, inflammation, synaptic pathology, and mitochondrial dysfunction, align closely with the complex nature of optic nerve degeneration. Careful preclinical validation and translational development could position psychoplastogens as valuable additions to future neuroprotective strategies, ultimately contributing to long-term preservation of visual function.

## Data Availability

No new data were created or analyzed in this study.

## Abbreviations

The following abbreviations are used in this manuscript:

(R)-DOI	2,5-dimethoxy-4-iodoamphetamine
5-Meo-DMT	5-methoxy-dimethyltryptamine
AMPA	α-Amino-3-hydroxy-5-methyl-4-isoxazolepropionic acid
BDNF	Brain-derived neurotrophic factor
CCB	Calcium channel blockers
CNS	Central nervous system
DMT	N,N-Dimethyltryptamine
fMRI	functional magnetic resonance
IEG	Immediate early gene
IL	Interleukin
IOP	Intraocular pressure
LSD	Lysergic acid diethylamide
MDMA	3,4-methylenedioxymethamphetamine
MMP	Matrix metalloproteinases
MSC	Mesenchymal stem cells
mTOR	Mammalian target of rapamycin
NFκB	Nuclear factor κB
NGF	Nerve Growth Factor
NTG	Normotensive glaucoma
OBF	Ocular blood flow
PET	Positron emission tomography
PKC	Protein kinase C
RGC	Retinal ganglion cell
ROS	Reactive oxygen species
Sig1R	Sigma-1 receptor
TBG	Tabernanthalog
TNF-α	Tumor necrosis factor α
TrkB	Tyrosine receptor kinase B
